# Bruch´s membrane thickness in relationship to axial length

**DOI:** 10.1371/journal.pone.0182080

**Published:** 2017-08-02

**Authors:** Hai Xia Bai, Ying Mao, Ling Shen, Xiao Lin Xu, Fei Gao, Zhi Bao Zhang, Bin Li, Jost B. Jonas

**Affiliations:** 1 Beijing Institute of Ophthalmology, Beijing Tongren Eye Center, Beijing Tongren Hospital, Capital Medical University, Beijing Ophthalmology & Visual Sciences Key Laboratory, Beijing, China; 2 Department of Ophthalmology, The 2nd Affiliated Hospital, Harbin Medical University, Harbin, Heilong-jiang, China; 3 Department of Ophthalmology, Medical Faculty Mannheim, Heidelberg University, Mannheim, Germany; National Eye Institute, UNITED STATES

## Abstract

**Purpose:**

To assess a potential role of Bruch´s membrane (BM) in the biomechanics of the eye, we measured its thickness and the density of retinal pigment epithelium (RPE) cells in various ocular regions in eyes of varying axial length.

**Methods:**

Human globes, enucleated because of an ocular tumor or end-stage glaucoma were prepared for histological examination. Using light microscopy, the histological slides were histomorphometrically examined applying a digitized image analysis system.

**Results:**

The study included 104 eyes with a mean axial length of 27.9±3.2 mm (range:22.6mm-36.5mm). In eyes without congenital glaucoma, BM was significantly thickest (*P*<0.001) at the ora serrata, followed by the posterior pole, the midpoint between equator and posterior pole (MBEPP), and finally the equator. BM thickness was not significantly correlated with axial length (ora serrata: *P* = 0.93; equator:*P* = 0.31; MBEPP:*P* = 0.15; posterior pole:*P* = 0.35). RPE cell density in the pre-equatorial region (*P* = 0.02; regression coefficient r = -0.24) and in the retro-equatorial region (*P* = 0.03; r = -0.22) decreased with longer axial length, while RPE cell density at the ora serrata (*P* = 0.35), the MBEPP (*P* = 0.06; r = -0.19) and the posterior pole (*P* = 0.38) was not significantly correlated with axial length. Highly myopic eyes with congenital glaucoma showed a tendency towards lower BM thickness and lower RPE cell density at all locations.

**Conclusions:**

BM thickness, in contrast to scleral and choroidal thickness, was independent of axial length in eyes without congenital glaucoma. In association with an axial elongation associated decrease in the RPE cell density in the midperiphery, the findings support the notion of a biomechanical role BM may play in the process of emmetropization/myopization.

## Introduction

Bruch´s membrane (BM) forms the border between the intravitreal cavity with the vitreous body and retina on its inner side and the choroid on its outer side [1]. It is formed by the retinal pigment epithelium (RPE) and consists of the basal membrane of the latter, two collagenous layers separated from each other by an elastic layer in its center, and the basal membrane of the choriocapillaris on its outer side. Together with the RPE, BM participates in forming the blood retina barrier, separating the retina with its almost fluid-free interstitial space from the spongy choroid with fenestrated choriocapillaris vessel walls and pronounced interstitial fluid. Recent studies on the emmetropization of the eye have suggested that BM, in addition to having a function in separating the retinal and vitreal compartment from the choroidal space, could play a biomechanical role in the process of emmetropization and myopization [2–9]. Since the biomechanical characteristics of a tissue strongly depend on its thickness, we measured the thickness of BM in different locations of human enucleated eyes. We also assessed the density of the RPE cells since the latter produce the BM.

## Methods

The histomorphometric investigation included human globes which had been enucleated due to uveal melanomas or end-stage painful glaucoma. The Medical Ethics Committee of the Beijing Tongren Hospital approved the study protocol according to the Declaration of Helsinki. The necessity of a written informed consent by the patients was waived since the eyes had been enucleated up to 50 years before the study was started. As inclusion criterion, all histological slides examined in the study had to run through the center of the cornea, the optic disc and through the posterior pole. Exclusion criteria were tissue changes attributable to the underlying diseases and which prevented a thickness measurement of BM.

**Fig 1:**
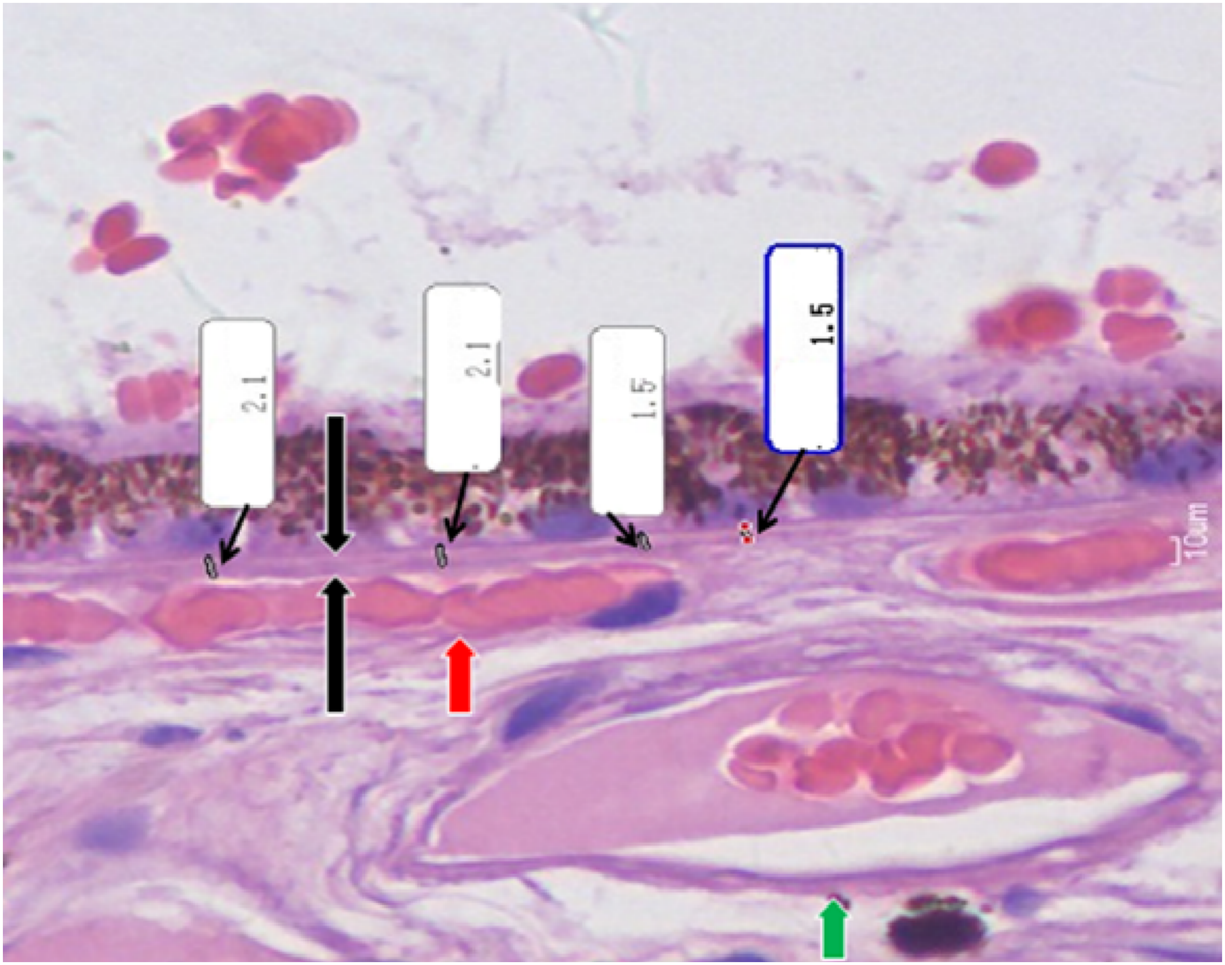
Histo-photograph showing thickness measurements of Bruch´s membrane (in μm); Black arrows: Bruch´s membrane; Red arrow: Choriocapillaris vessel; Green Arrow: Large choroidal vessel.

After processing the enucleated eyes were prepared in a standardized manner for light microscopical examination. It included fixation in a solution of 4% formaldehyde for at least 24 hours, measurement of the sagittal globe diameter, sectioning of the eyes, and staining of the slides with hematoxycilin eosin or by the Periodic-Acid-Shiff (PAS) method. The slides were histomorphometrically examined using a digitized image analysis system (Moticam 2006 (2.0M pixel USB2.0) and Motic Digital Medical Image Analysis System, Motic China group, Co. Ltd. Xiamen, China). We measured the thickness of BM (defined as the distance between the basal membrane of the RPE on its inner side to the basal membrane of the choriocapillaris on its outer side), the thickness of the choroid and the thickness of the sclera at the ora serrata, the equator, the midpoint between the equator and the posterior pole (MBEPP), and at the posterior pole (Fig 1). Scleral thickness was additionally determined in the pars plana region. At each measurement region, four measurements at slightly different points were obtained and the mean of these measurements was taken for further statistical analysis. For the assessment of the density of the RPE cells, we used the same digitized image analysis system, and we counted the number of RPE cells on Bruch´s membrane for a length of 480 μm in the region just posterior to the ora serrata, the region just anterior to the equator, the region just posterior to the equator, at the MBEPP, and at the posterior pole. The methods have been described in detail recently [4–8,10].

A statistical analysis program (SPSS, version 22.0, IBM-SPSS, Chicago, IL, USA) was used for the statistical analysis. In a first step, we calculated the mean values ± standard deviations of the outcome parameters at each measurement location. Using a two-tailed student-t-test for paired samples, we then compared in a second step the BM thickness measurements and RPE density values between the various locations of measurement. Finally, in linear regression analysis, we performed a univariate analysis, followed by a multivariate analysis, to test associations between BM thickness or the RPE cell count with axial length and other parameters. All *P*-values were 2-sided and considered statistically significant when less than 0.05.

## Results

The study included 104 globes of 104 patients (51 women) with a mean age of 35.7 ± 18.4 years (median: 35 years; range:1–81 years) and a mean axial length of 27.9 ± 3.2 mm (median: 27.5 mm; range: 22.6mm–36.5mm). Reasons for enucleation were uveal melanoma in 34 patients and end-stage painful glaucoma in 70 patients, among them five patients with congenital glaucoma. Axial length was longer than 26.0 mm in 64 eyes.

**Fig 2:**
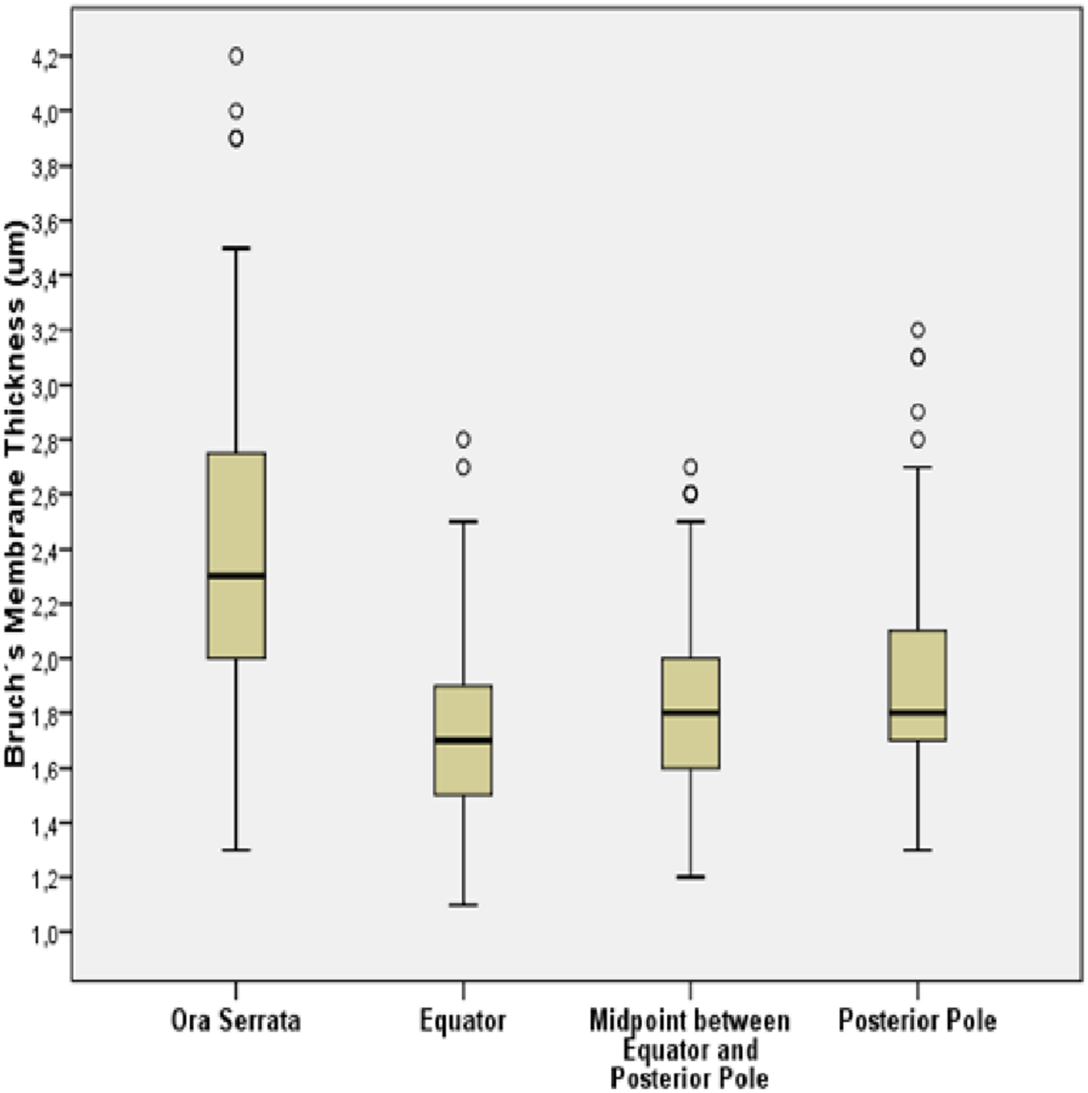
Graph showing the distribution of Bruch´s membrane thickness at various ocular locations in enucleated human eyes with globes with congenital glaucoma excluded.

**Fig 3:**
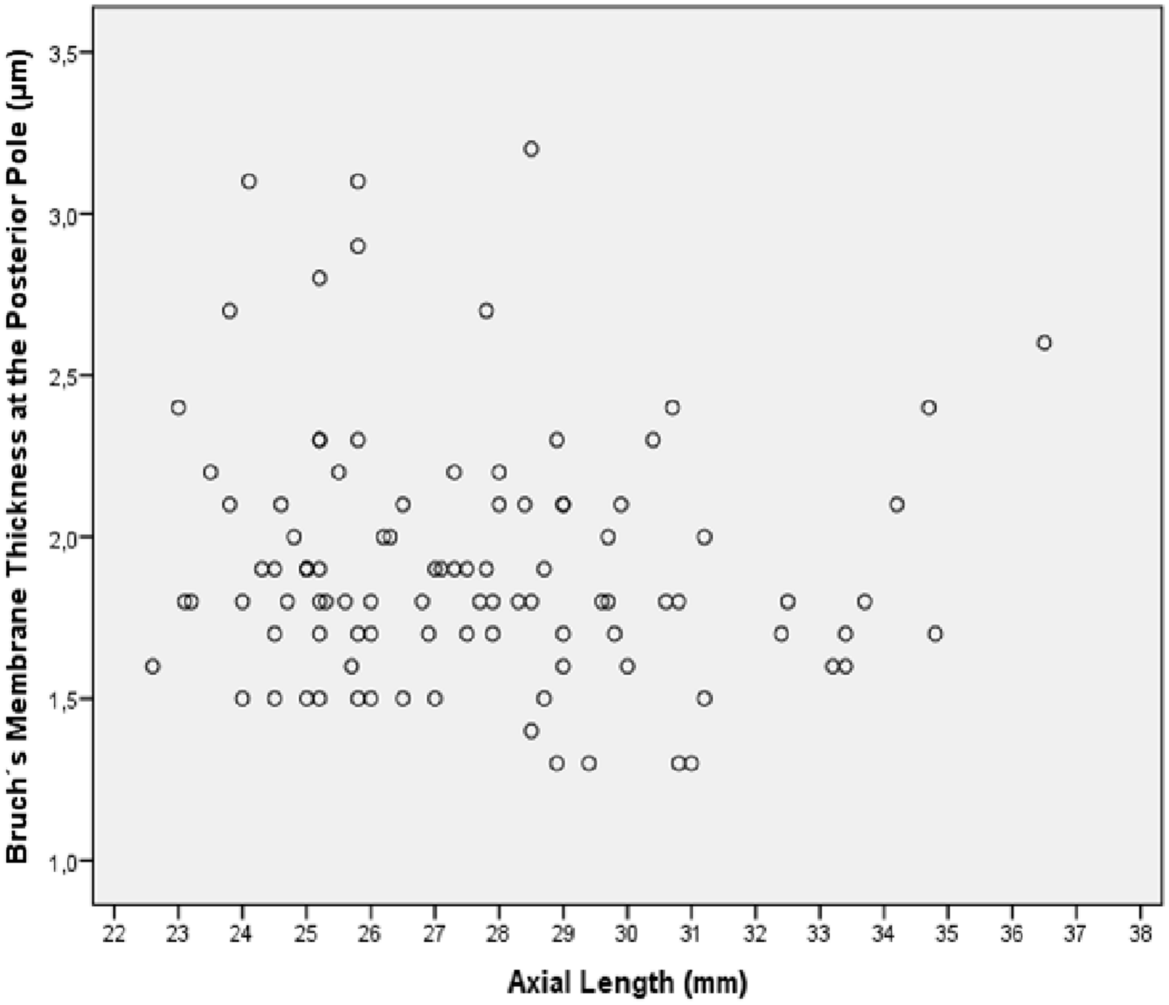
Graph showing the distribution of Bruch´s membrane thickness at the posterior pole versus axial length in enucleated human eyes without congenital glaucoma.

In the eyes without congenital glaucoma, BM thickness was significantly the thickest (*P*<0.001) at the ora serrata, followed by the posterior pole, the MBEPP, and the equator (Table 1) (Fig 2). In the eyes without congenital glaucoma, BM thickness was not significantly correlated with axial length, neither at the ora serrata (*P* = 0.93), the equator (*P* = 0.31), the MBEPP (*P* = 0.15) nor the posterior pole (*P* = 0.35) (Fig 3). Correspondingly, the non-highly myopic group and the highly myopic group without congenital glaucoma did not differ significantly (*P*>0.15) in BM thickness (Table 1). BM thickness showed a tendency towards thinner measurements in the group of eyes with secondary high myopia due to congenital glaucoma as compared to both other groups (Table 1). The differences were however not statistically significant.

**Table 1:** Histomorphometric measurements in enucleated human globes (Mean ± Standard Deviations).

Parameter	Total Group	Non-Highly Myopic Group	Highly Myopic Group Without Congenital Glaucoma	*P*-Value (1)	Congenital Glaucoma (Secondary High Myopia)	*P*-Value (2)
N	104	40	59		5	
Age (Years)	35.7 ± 18.4	44.7 ± 15.3	31.8 ± 17.5	0.15	9.4 ± 8.7	0.15
Axial Length (mm)	27.9 ± 3.2	24.8 ± 0.9	29.5 ± 2.4	<0.001	33.6 ± 1.7	0.10
Bruch´s Membrane Thickness at:						
Ora Serrata	2.4 ± 0.6	2.4 ± 0.6	2.3 ± 0.5	0.26	2.0 ± 0.7	0.86
Equator	1.7 ± 0.3	1.7 ± 0.3	1.7 ± 0.3	0.76	1.5 ± 0.2	0.46
Midpoint between Equator and Posterior Pole	1.8 ± 0.3	1.8 ± 0.3	1.8 ± 0.3	0.92	1.9 ± 0.3	0.50
Parapapillary Region	2.0 ± 0.4	2.0 ± 0.3	2.0 ± 0.4	0.34	1.7 ± 0.2	0.12
Posterior Pole	1.9 ± 0.4	2.0 ± 0.4	1.9 ± 0.4	0.16	1.8 ± 0.3	0.57
Choroidal Thickness at:						
Ora Serrata	34.9 ± 12.2	37.9 ± 14.4	33.3 ± 10.6	0.06	31.5 ± 9.3	0.67
Equator	32.3 ± 14.1	36.9 ± 16.0	29.5 ± 12.2	0.09	28.8 ± 13.1	0.86
Midpoint between Equator and Posterior Pole	53.9 ± 24.0	61.2 ± 22.0	49.5 ± 24.4	0.64	46.7 ± 21.5	0.92
Parapapillary Region	52.7 ± 27.1	60.6 ± 32.4	49.3 ± 21.6	0.12	28.7 ± 19.3	0.54
Posterior Pole	67.1 ± 34.5	80.5 ± 40.0	60.1 ± 27.2	0.09	45.3 ± 37.3	0.93
Scleral Thickness at:						
Pars Plana	453 ± 90	494 ± 81	435 ± 85	0.81	340 ± 54	0.26
Ora Serrata	407 ± 102	445 ± 107	387 ± 91	0.17	329 ± 83	0.48
Equator	360 ± 77	366 ± 79	359 ± 77	0.72	314 ± 57	0.32
Midpoint between Equator and Posterior Pole	480 ± 114	501 ± 99	467 ± 116	0.26	463 ± 192	0.13
Parapapillary Region	718 ± 190	822 ± 138	667 ± 185	0.02	472 ± 142	0.35
Posterior Pole	722 ± 186	813 ± 117	679 ± 194	0.001	491 ± 175	0.82
Retinal Pigment Epithelium Cell Density						
Posterior to the Ora Serrata	22.8 ± 2.6	23.0 ± 2.7	22.6 ± 2.6	0.77	22.4 ± 2.9	0.90
Anterior to the Equator	21.5 ± 2.5	22.1 ± 2.2	21.2 ± 2.1	0.50	21.4 ± 2.2	0.49
Posterior to the Equator	21.5 ± 2.2	21.9 ± 2.0	21.3 ± 2.4	0.44	21.6 ± 1.9	0.66
Midpoint between Equator and Posterior Pole	23.8 ± 2.4	24.1 ± 1.7	23.7 ± 2.7	0.025	22.0 ± 3.2	0.35
Posterior Pole	27.0 ± 3.0	27.3 ± 1.9	27.0 ± 3.5	0.02	24.8 ± 3.3	0.63

*P*-Value (1): Statistical significance of the difference between the non-highly myopic group and the highly myopic group without congenital glaucoma

*P*-Value (2): Statistical significance of the difference between the congenital glaucoma group and the remaining eyes

**Fig 4:**
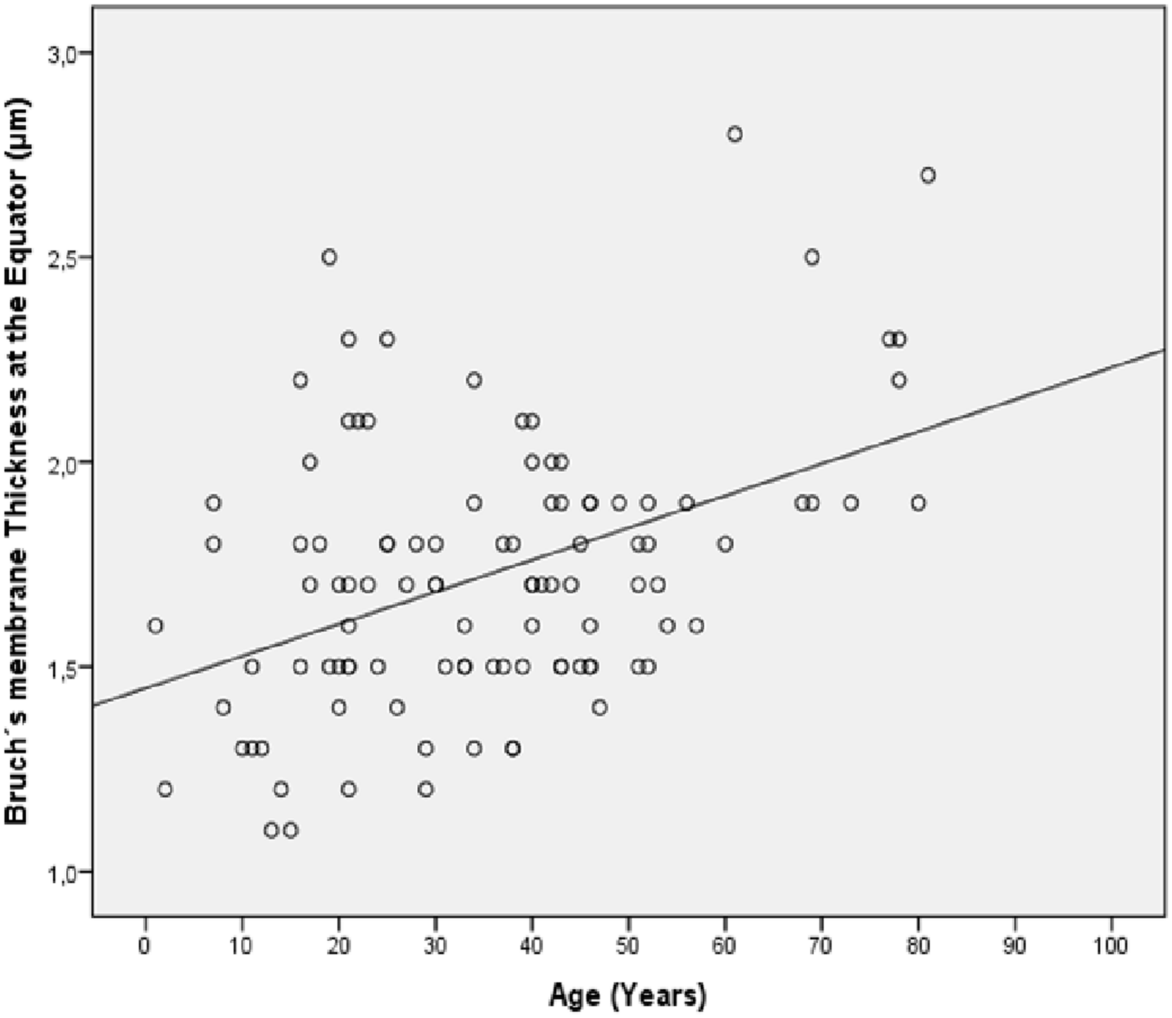
Graph showing the distribution of Bruch´s membrane thickness at the equator versus age in enucleated human eyes (Equation of the regression line: Bruch´s membrane thickness at the Equator (μm) = 0.008 x Age (Years) + 1.45).

BM thickness increased significantly with older age when measured at the ora serrata (*P* = 0.01; r = 0.25) and at the equator (*P*<0.001; r = 0.43), while BM thickness measured at the MBEPP (*P* = 0.20) or at the posterior pole (*P* = 0.17) was not significantly associated with age (Fig 4).

BM thickness measurements obtained at any location did not differ between the glaucomatous group and the non-glaucomatous group (*P*>0.30).

**Fig 5:**
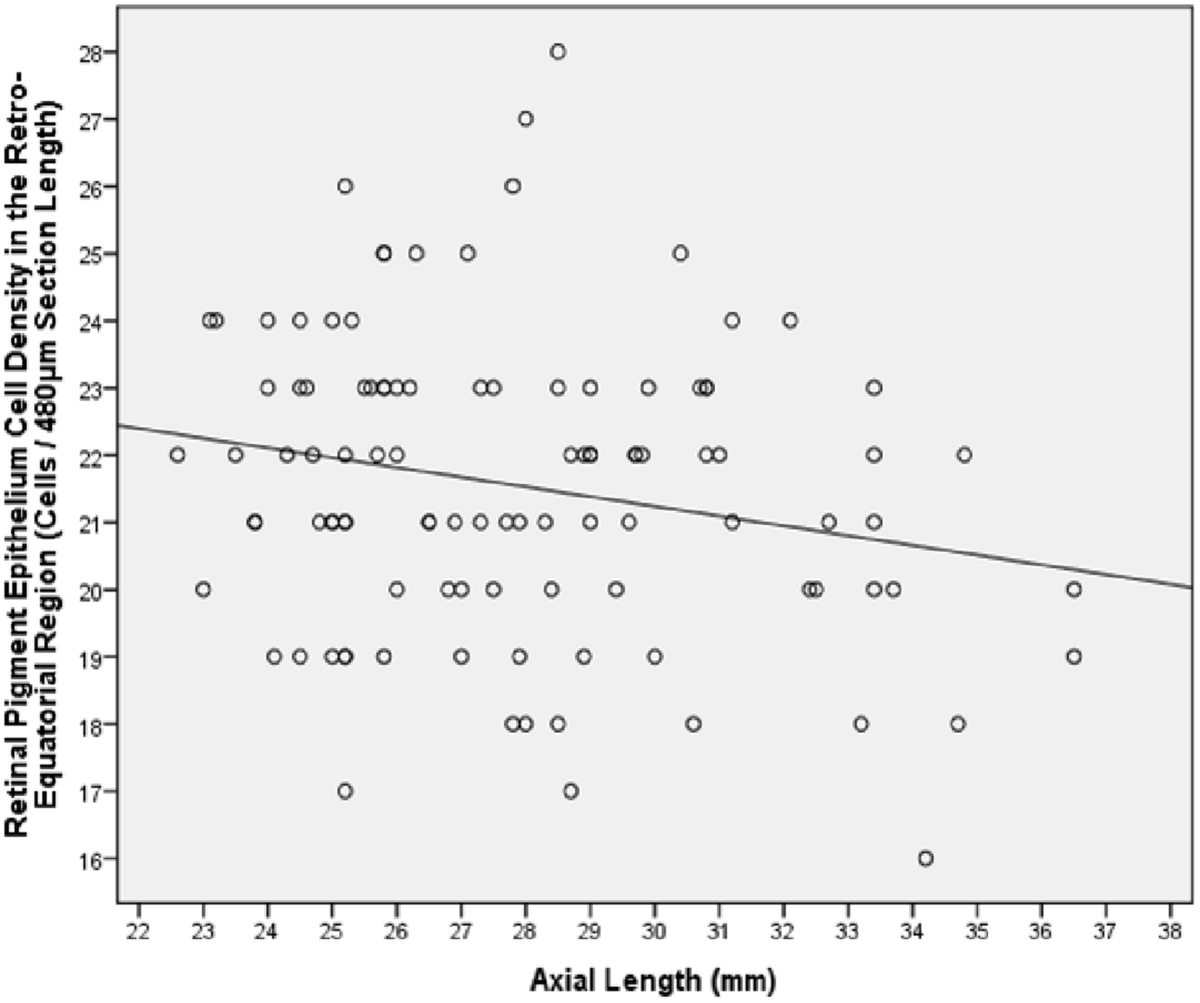
Graph showing the distribution of the retinal pigment epithelium cell density at the posterior pole versus axial length in enucleated human eyes without congenital glaucoma (Equation of the regression line: RPE Cell density (Cells / 480μm Section Length)) = -0.15 x Axial Length (mm) + 25.6).

**Fig 6:**
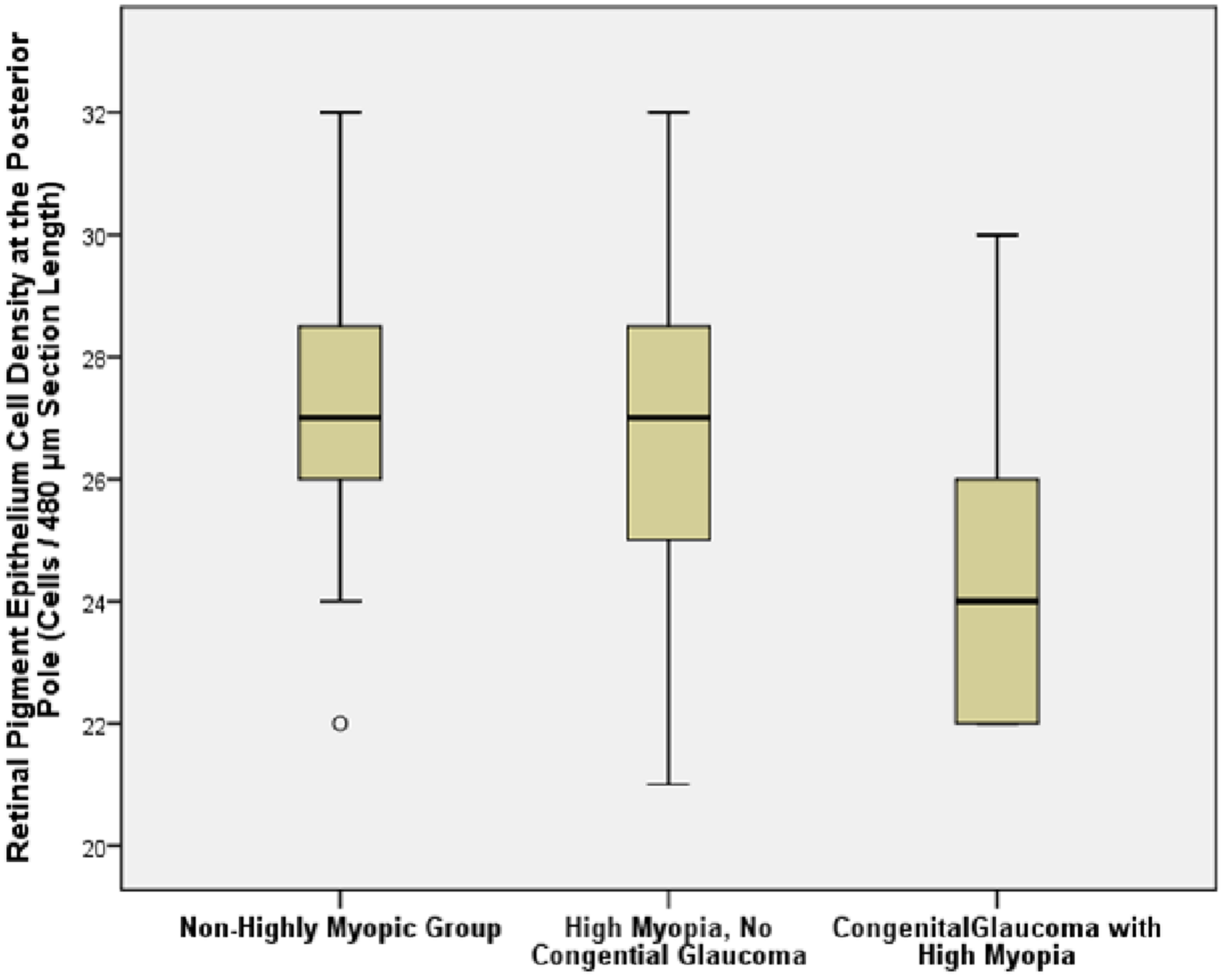
Graph showing the distribution of the retinal pigment epithelium cell density at the posterior pole (cells / 480μm section length) in enucleated human eyes, stratified by high myopia and presence of congenital glaucoma.

In the eyes without congenital glaucoma, RPE cell density measured in the pre-equatorial region (*P* = 0.02; regression coefficient r = -0.24) and in the retro-equatorial region (*P* = 0.03; r = -0.22) decreased with longer axial length, while RPE cell density in the ora serrata region (*P* = 0.35), at the MBEPP (*P* = 0.06; r = -0.19) and at the posterior pole (*P* = 0.38) was not significantly correlated with axial length (Figs 5 and 6). RPE density at the posterior pole showed a tendency to be lower in the eyes with secondary high myopia due to congenital glaucoma (24.8 ± 3.3 cells / 480μm section length) than in the eyes with primary high myopia (27.0 ± 3.5 cells / 480μm section length) and the non-highly myopic eyes (27.0 ± 3.0 cells / 480μm section length) (Table 1) (Fig 6). The differences were however not statistically significant (*P* = 0.20).

**Fig 7:**
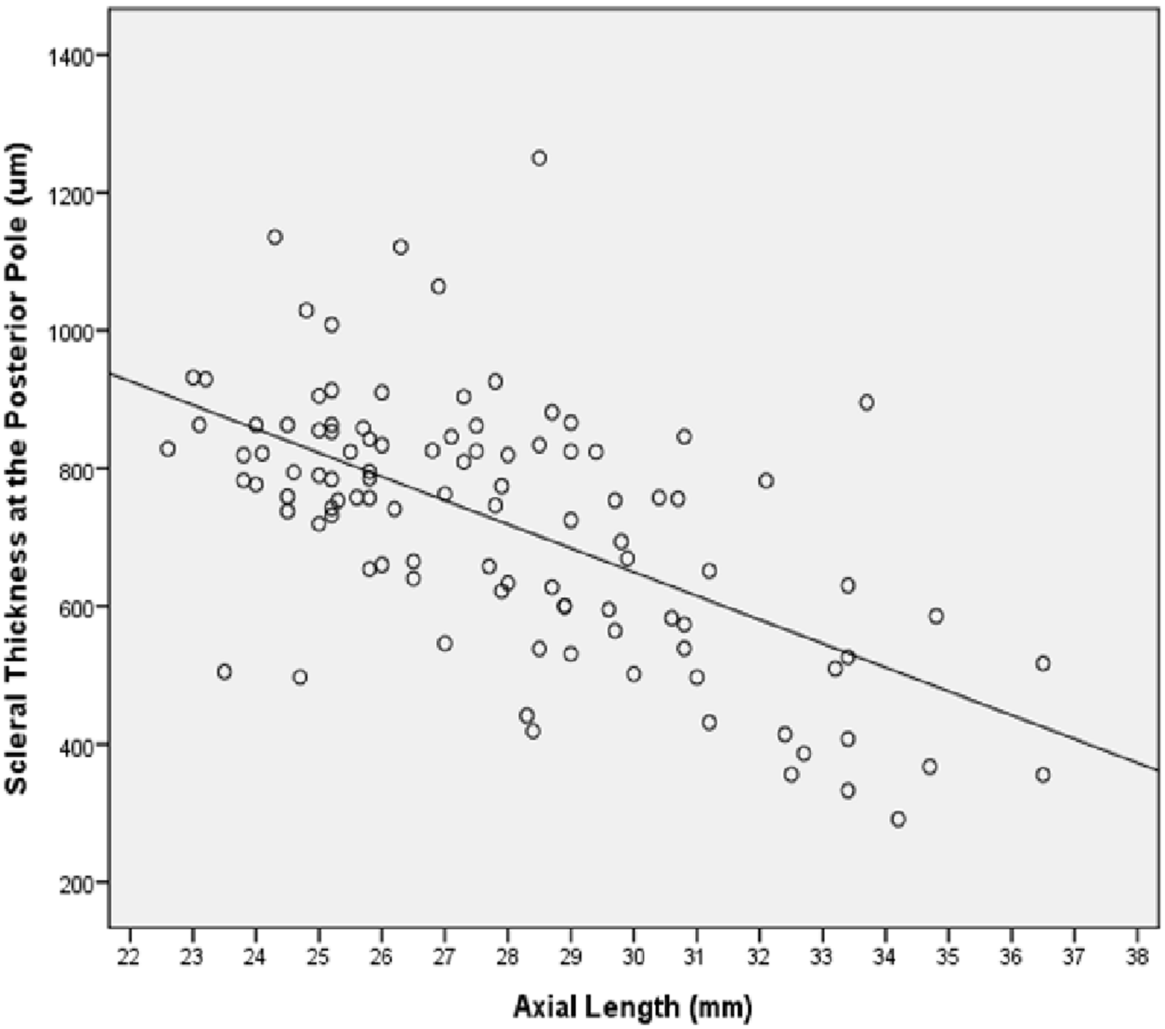
Scatterplot showing the association between scleral thickness at the posterior pole and axial length in enucleated human eyes (Equation of the regression line: Scleral thickness at Posterior Pole (μm) = -34.6 x Axial Length (mm) + 1688).

The thickness of the choroid decreased significantly with longer axial length for choroidal thickness measurements obtained at the equator (*P* = 0.006; r = -0.27), at the MBEPP (*P*<0.001; r = -0.35) and at the posterior pole (*P*<0.001; r = -0.39). The association was most marked for the choroidal measurements taken at the posterior pole. There was no difference if the highly myopic eyes with congenital glaucoma were included or excluded from the analysis. Scleral thickness measured at the ora serrata (*P*<0.001; r = -0.51), the equator (*P*<0.001; r = -0.13), the MBEPP (*P* = 0.02; r = -0.23) and at the posterior pole (*P*<0.001; r = -0.60) decreased with longer axial length, most marked at the posterior pole (Fig 7). Again, there was no difference if the highly myopic eyes with congenital glaucoma were included or excluded from the analysis.

## Discussion

In this histomorphometric study on human eyes, the thickness of BM was not related with axial length in eyes without congenital glaucoma. The density of the RPE cells decreased with longer axial length in the pre-equatorial and retro-equatorial region, while at the posterior pole, as was the thickness of BM, the RPE cell density was not significantly correlated with axial length. In contrast, thickness of choroid and of the sclera markedly decreased with longer axial length, most marked at the posterior pole.

These findings obtained on Chinese eyes confirmed the results of a previous investigation on Western European eyes, in which as in the present study, BM thickness was not related with axial length in eyes without congenital glaucoma, and in which the RPE cell density decreased in the retro-equatorial region with elongating axial length [8,10]. In eyes with congenital glaucoma examined previously, BM thickness showed a marginally significant inverse association with axial length, while the present study only showed a tendency towards a thinner BM in the highly myopic eyes with congenital glaucoma [6].

The findings obtained in the present study and in the previous investigation on different study populations cannot fully be compared with results obtained in other studies, since BM thickness has not intensively been examined yet, except for in a relatively few studies on BM thickness in relation to age-related macular degeneration [11–17]. In all these latter studies, BM thickness was not compared with axial length.

The BM thickness measurements obtained in the present study were thinner than in the previous study in which BM thickness was measured with the help of a millimeter scale aligned with the ocular of the light microscope [10]. The values of the present study were also slightly thinner than the measurements of BM thickness Ramrattan and colleagues obtained with values ranging between 2 and 5 μm [13]. Reason for the discrepancy between both studies might have been that Ramrattan´s study in contrast to our investigation also included eyes with age-related macular degeneration.

The finding that BM thickness, in contrast to choroidal thickness and scleral thickness, did not decrease with increasing axial length fitted with the hypothesis that BM might play a role in the process of emmetropization [2]. The latter, taking place after the end of the second year of life, has been defined as the adjustment of the length of the optical axis in relationship to the refractive power of cornea and lens. The hypothesis was based on findings that after a spherical eye growth with active increase in scleral volume till the end of the second year of life, the dimensions of cornea and lens remain mostly constant while the globe elongates axially to bring the foveola into the optical focus. That axial elongation was accompanied by a thinning of the choroid and sclera, leading to a re-arrangement of the available choroidal and scleral tissue without a major active increase in tissue volume [4,18,19]. Thinning of the choroid and sclera was most marked at the posterior pole. In contrast to the choroidal and scleral thinning, retinal thickness in the macular region did not decrease with longer axial length, parallel to the observation that best corrected visual acuity was independent of axial length if eyes with myopic maculopathy were excluded [7,20]. There was however a thinning of the retina in the midperiphery collateral to a decrease in the density of the RPE cells in the same region, as has also been found in the present study [7,8]. Other studies had suggested that the detection of a defocus of the image on the retina is perceived in the retro-equatorial region [21–24]. Since BM thickness was independent of axial elongation as found in the present study and the previous investigation on an ethnically different study population, it has been postulated that axial elongation occurred by a production of new BM in the retro-equatorial region. It could explain the axial elongation-associated thinning of the retina and decrease in the density of the RPE cells in that region. It could also explain that the length of BM in the macular region, the density of the RPE cells at the posterior pole (as also shown in the present study), the macular retinal thickness, and correspondingly, best corrected visual acuity, were independent of axial length. The axial elongation-related increase in the optic disc-fovea distance occurred by the development and enlargement of parapapillary gamma zone without BM [25]. Fitting with the hypothesis of BM playing a role in axial elongation, a recent experimental study on guinea pigs with lens-induced myopization showed that an intravitreally applied antibody of amphiregulin was associated with a dosage-dependent decrease in axial elongation [9]. Amphiregulin is a member of the epithelial growth factor family, and the RPE has receptors for epidermal growth factor and amphiregulin [9,26].

If the results of the present study are discussed, potential limitations should be taken into account. First, the measurements were influenced by the post mortem tissue swelling and fixation induced tissue shrinkage. Since BM does not contain blood vessels and may not show a marked edema, it might have been unlikely however, that preparation-associated tissue changes had markedly changed BM thickness. Second, the study material consisted of sagittal sections through the pupil and the optic nerve, while serial sections of the eyes were not available. Third, the investigation consisted of eyes with tumors or end-stage glaucoma so that it has remained unclear whether the results can be transferred onto normal human eyes. Fourth, the group of eyes with congenital glaucoma was rather small with 5 globes included, so that the statistical power for analysis of the data of these eyes was limited.

In conclusion, BM thickness, in contrast to scleral and choroidal thickness was independent of axial length in eyes without congenital glaucoma. In association with an axial elongation associated decrease in the RPE cell density in the midperiphery, the findings support the notion of a biomechanical role BM may play in the process of emmetropization/myopization.

## Supporting information

S1 Datafile S1 datafile containing microdata of the study. (SAV)
